# The transcriptome of the rumen ciliate *Entodinium caudatum* reveals some of its metabolic features

**DOI:** 10.1186/s12864-019-6382-x

**Published:** 2019-12-21

**Authors:** Lingling Wang, Anas Abu-Doleh, Johanna Plank, Umit V. Catalyurek, Jeffrey L. Firkins, Zhongtang Yu

**Affiliations:** 1 Department of Animal Sciences, The Ohio State University, 2029 Fyffe Court, Columbus, OH 43210 USA; 2 Department of Biomedical Informatics, The Ohio State University, Columbus, OH USA; 3 Department of Electrical and Computer Engineering, The Ohio State University, Columbus, OH USA; 4 Current address: Department of Biomedical Systems and Informatics Engineering, Yarmouk University, Irbid, Jordan; 5 Current address: School of Computational Science and Engineering, Georgia Institute of Technology, Atlanta, GA USA

**Keywords:** *Entodinium caudatum*, Metabolism, RNA-Seq, Rumen protozoa, Transcriptomics

## Abstract

**Background:**

Rumen ciliates play important roles in rumen function by digesting and fermenting feed and shaping the rumen microbiome. However, they remain poorly understood due to the lack of definitive direct evidence without influence by prokaryotes (including symbionts) in co-cultures or the rumen. In this study, we used RNA-Seq to characterize the transcriptome of *Entodinium caudatum*, the most predominant and representative rumen ciliate species.

**Results:**

Of a large number of transcripts, > 12,000 were annotated to the curated genes in the NR, UniProt, and GO databases. Numerous CAZymes (including lysozyme and chitinase) and peptidases were represented in the transcriptome. This study revealed the ability of *E. caudatum* to depolymerize starch, hemicellulose, pectin, and the polysaccharides of the bacterial and fungal cell wall, and to degrade proteins. Many signaling pathways, including the ones that have been shown to function in *E. caudatum*, were represented by many transcripts. The transcriptome also revealed the expression of the genes involved in symbiosis, detoxification of reactive oxygen species, and the electron-transport chain. Overall, the transcriptomic evidence is consistent with some of the previous premises about *E. caudatum*. However, the identification of specific genes, such as those encoding lysozyme, peptidases, and other enzymes unique to rumen ciliates might be targeted to develop specific and effective inhibitors to improve nitrogen utilization efficiency by controlling the activity and growth of rumen ciliates. The transcriptomic data will also help the assembly and annotation in future genomic sequencing of *E. caudatum*.

**Conclusion:**

As the first transcriptome of a single species of rumen ciliates ever sequenced, it provides direct evidence for the substrate spectrum, fermentation pathways, ability to respond to various biotic and abiotic stimuli, and other physiological and ecological features of *E. caudatum*. The presence and expression of the genes involved in the lysis and degradation of microbial cells highlight the dependence of *E. caudatum* on engulfment of other rumen microbes for its survival and growth. These genes may be explored in future research to develop targeted control of *Entodinium* species in the rumen. The transcriptome can also facilitate future genomic studies of *E. caudatum* and other related rumen ciliates.

## Background

Rumen protozoa are strictly anaerobic and highly specialized ciliates that can survive only in the rumen and similar habitats [1]. These ciliates play important roles in feed utilization and impact the environmental footprint (methane emission and nitrogen excretion) of ruminant livestock production [2, 3]. Although numerically much less abundant than rumen bacteria, rumen ciliates account for a large portion of the total microbial biomass due to their large cell size. In the rumen of domesticated cattle and sheep, rumen ciliates collectively account for 20 to 50% of the total microbial biomass [4]. Throughout millions of years of evolution, rumen ciliates developed symbiotic relationships with their animal hosts and both symbiotic and predator-prey relationships with other members of the rumen microbiota. Researchers began to study rumen ciliates in the 1950s [5, 6] and made repeated attempts to establish axenic cultures (a culture free of bacteria, archaea, and fungi) of individual rumen ciliate species to definitively characterize their metabolism, physiology, and ecology. However, no one has succeeded in establishing an axenic culture of any rumen ciliate species that can be maintained long enough (typically no longer than a week) for research [7–9]. The lack of axenic cultures of rumen ciliates has forced researchers to utilize other methods to infer metabolism and functions of rumen protozoa, such as comparing the rumen fermentation and microbial profiles of faunated and defaunated (ciliate free) cattle or sheep, or using in vitro cultures of washed rumen ciliate cells, which still contained unknown (both taxonomically and quantitatively) prokaryotic species. Because of the unknown confounding factors, such as variations of rumen microbiome in the absence or presence of protozoa and potential prokaryotic contamination, the fundamental biological characteristics of rumen protozoa remain to be definitively determined. For example, their substrate spectrum, fermentation products, metabolic pathways, recruitment of symbionts, and prey selection all remain to be fully elucidated. As another example, rumen ciliates are thought to scavenge O_2_ that enters the rumen (together with the ingested feed, drinking water, saliva, and perfusion from the rumen wall), thereby protecting strictly anaerobic archaea and bacteria, particularly cellulolytic bacteria [10]. However, it remains to be determined if and how rumen ciliates utilize O_2_.

Transcriptomics is a powerful tool to reveal the genes expressed in an organism and thus enables characterization of its metabolism and other biological processes and features. Before next-generation sequencing (NGS) technologies became available, the first transcriptomic study of ciliates used sequencing analysis of expressed sequence tags (ESTs) to assess the gene expression of model ciliate *Tetrahymena thermophila* [11]. Through genome-scale gene discovery and functional analysis, that study greatly advanced the understanding of the biological features of *T. thermophila*. Additionally, it revealed that 11% of the non-*Tetrahymena* specific genes were present in humans and other mammals but not found in other model unicellular eukaryotes, reinforcing the status of *Tetrahymena* as an excellent model for studying many aspects of animal biology. The transcriptome of *T. thermophila,* determined recently using RNA-Seq, provided a fully comprehensive view of its global gene expression [12] and significantly improved its genome annotation [12, 13]. *Plasmodium falciparum*, the protozoan parasite that causes malaria in humans, has been subjected to repeated transcriptomic studies using all the available technologies or approaches, including DNA microarrays [14], cDNA libraries [15], serial analysis of gene expression (SAGE) [16], and RNA-Seq [17]. These studies enabled a comprehensive understanding of the biological features at each stage of its life cycle, identification of gene targets for drug development, and discoveries of drug resistance mechanisms in *P. falciparum* [18, 19].

Three transcriptomic studies have been reported on rumen ciliates. The first study analyzed only a small number of ESTs from 10 species of rumen ciliates [20], and two recent studies analyzed the eukaryotic (both ciliates and fungi) transcripts of an entire ruminal microbiota using a metatranscriptomic approach [21, 22]. These studies provided direct evidence of some metabolic features of rumen ciliates and suggested the high likelihood of horizontal gene transfers (HGT). However, the small number of transcripts determined only revealed a tip of the complex biological iceberg of rumen ciliates. The objectives of the present study were to discover the genes of *Entodinium caudatum*, a predominant rumen ciliate species, and to gain a better understanding of its metabolism and physiological and ecological characteristics. We used RNA-Seq to analyze a clonal ciliate monoculture of *E. caudatum* MZG-1 as the only ciliate. We found more than 33,000 transcripts that provided new insights into the metabolic and other biological features of *E. caudatum*.

## Results

### Overview of the *Entodinium caudatum* transcriptome

From nearly 60 million raw sequencing reads, approximately 21.6 million sequences resulted after filtering with a Q score ≥ 30 and joining of the paired reads (Additional file 1: Table S1). De novo assembly of the quality-checked sequences using Trinity [23] resulted in 58,899 contigs. After filtering out the contigs with low coverage (less than 5×), putative contaminations of prokaryotic transcripts, and other uncertain sequences, 33,546 contigs (referred to as transcripts hereafter) remained, with an average length of 759 bases and N50 of 596 bases. About 54% of the transcripts had low sequence similarity with any of the sequences in the NR or UniProt databases. The relative abundance (% of total transcripts) of each unique transcript varied considerably. The transcripts at the highest abundance were annotated to coding for proteins involved in cellular structures and processes that are essential to eukaryotic cells (Additional file 2: Table S2). These include (i) histone proteins, such as macronuclear histone; (ii) cell motor and skeleton, such as actin, profilin, tubulin, dynein, and centrin; (iii) signal transduction proteins such as the 14–3-3 protein that binds to many functionally diverse proteins involved in signal transduction; (iv) protein translation; (v) carbohydrate metabolism enzymes such as pyruvate phosphate dikinase (PPDK); and (vi) nucleotide metabolism enzymes such as nucleoside-diphosphate kinase (NDPK). Transcripts annotated to code for proteolysis were also abundant, and these include polyubiquitin- and ubiquitin-conjugating enzymes, cysteine proteinase including cathepsins B and F, both of which are lysosomal cysteine peptidases, and cysteine protease inhibitors such as cystatin-B-like protein. Two of the highly expressed cysteine proteinases were annotated to having a signal peptide.

### The COG, GO, and KEEG classification of the *E. caudatum* transcripts

**Fig. 1:**
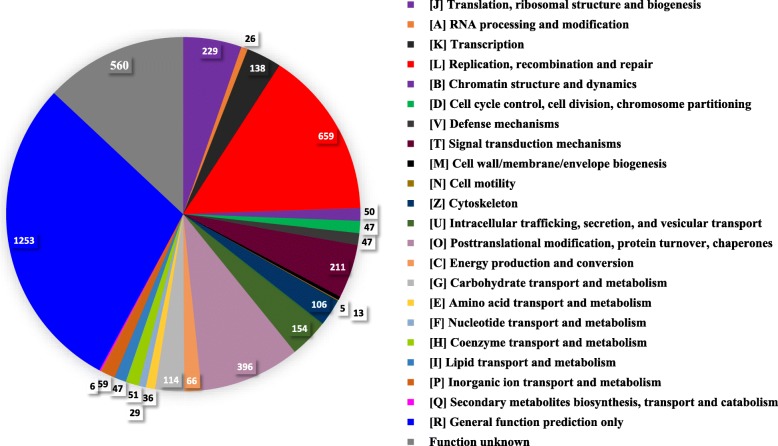
COG classification of the *E. caudatum* transcriptome

Comparison of the transcript sequences to the COG database using MEGAN5 [24] assigned 4302 different transcripts to all of the 23 COG functional categories (Fig. 1). The largest category was general function (Category R), followed by replication, recombination, and repair (Category L); function unknown (Category S); posttranslational modification, protein turnover, and chaperones (Category O); translation, ribosomal structure, and biogenesis (Category J); signal transduction (Category T); cytoskeleton (Category Z); intracellular trafficking, secretion, and vesicular transport (Category U); and carbohydrate transport and metabolism (Category G).

Of the 15,724 transcripts that each had an NR hit, 12,652 were assigned to 8665 non-redundant GO terms. Using the WEGO online tool (wego.genomics.org.cn), these transcripts were annotated to a large number of level-3 subcategories of cellular components, molecular function, and biological processes (Additional file 3: Table S3). Among the highly abundant transcripts annotated to level-3 subcategories of cellular components are cell parts (including intracellular parts, endomembrane systems, cell periphery, and plasma membrane), organelles and organelle parts (e.g., organelle membrane and lumen, membrane-bounded organelles, and non-membrane-bounded organelles), and protein-containing complexes. Other transcripts at high abundance were annotated to genes involved in cell projection parts, cell leading edge parts, apical parts of cells, clathrin-coated pits, cilium and ciliary parts, extracellular organelles and region parts, intraciliary transport particles, proteasome core complexes, proteasome regulatory particles, TOR complexes (both TORC1 and TORC2), and DNA packaging complexes. In the molecular function category, transcripts at high abundance were found encoding catalytic activities (e.g., hydrolases, transferases, oxidoreductases, catalytic activities acting on RNA, and ligases), binding (binding of organic cyclic and heterocyclic compounds, carbohydrate derivatives, small molecules, ions, proteins, lipids, and drugs), molecular function regulators (e.g., regulators of enzymes, guanyl-nucleotide exchange factor activities, and channels), molecular transducers (e.g., signal receptors, cyclin-dependent protein kinases, and cyclic nucleotide-dependent protein kinases), transporters (e.g., transmembrane transporters, lipid transporters, and protein transporters), structural molecules (e.g., protein-containing complex scaffolds, structural constituents of ribosomes, and structural constituents of cytoskeletons), and transcription regulators (DNA-binding transcription factors and transcription coregulators). The biological process has the largest number of transcripts annotated to level-3 subcategories. Among the highly expressed genes were the ones involved in cellular developmental processes, cellular processes (development, components, response, signal transduction, regulation, communication, cell cycle), cellular component organization or biogenesis, localization (establishment, maintenance, regulation), regulation (biological quality, processes, and molecular function), response to stimuli (stress, chemical, biotic, abiotic, external, endogenous, regulation), signaling (signal transduction and regulation, cell-cell signaling), regulation of biological processes, metabolic processes (organic, nitrogenous compounds, biosynthesis, catabolism, and regulation), regulation of biological processes (both positive and negative). One GO term (GO:0061783 peptidoglycan muralytic activity) involved in peptidoglycan degradation was also represented.

**Fig. 2:**
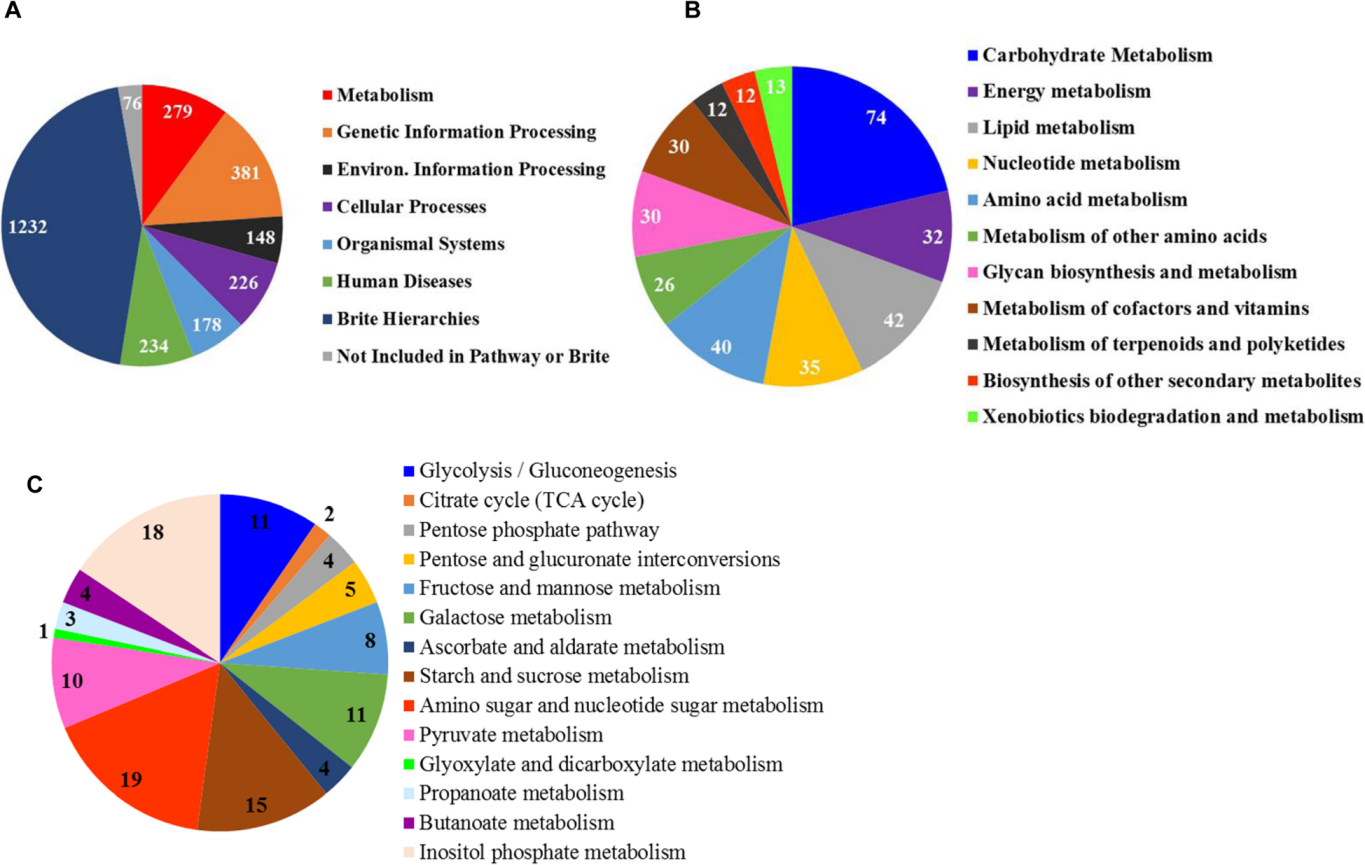
The KEGG classification of *E. caudatum* transcriptome at subsystem level_1 (**a**, overall), level_2 (**b**, metabolism), and level_3 (**c**, carbohydrate metabolism)

By comparing the transcript sequences to the KEGG database, 5598 transcripts were assigned to 1516 functional orthologs (KOs) and further mapped to 343 pathways involved in Cellular Processes (20.8% of total transcripts assigned to a KEGG class), Environmental Information Processing (20.4%), Genetic Information Processing (16.6%), Human Diseases (25.6%), Metabolism (12.6%), and Organismal Systems (22.8%) (Fig. 2a, Additional file 4: Table S4). About 250 of the transcripts related to metabolism could not be classified to a pathway or a BRITE (A KEGG BRITE is a collection of manually created hierarchical text (htext) files capturing functional hierarchies of various biological objects, especially those represented as KEGG objects). Within the metabolism category, carbohydrate metabolism was represented by the largest number of transcripts, followed by lipid metabolism, metabolism of cofactors and vitamins, and nucleotide metabolism (Fig. 2b, Additional file 4: Table S4). Of the transcripts involved in carbohydrate metabolism, inositol phosphate metabolism and starch and sucrose metabolism were abundantly represented, followed by galactose metabolism, amino sugar and nucleotide sugar metabolism, pyruvate metabolism, fructose and mannose metabolism, pentose and glucuronate interconversions, and glycolysis (Fig. 2c, Additional file 4: Table S4). The TCA cycle was only represented by two transcripts. Within the Genetic Information Processing category, spliceosome, mRNA surveillance, protein processing in the endoplasmic reticulum, ubiquitin-mediated proteolysis, and RNA degradation (besides ribosomes) were among the highly expressed categories. In the Environmental Information Processing category, 32 signaling pathways were represented by varying numbers of transcripts (detailed later in Transcripts involved in signal transductions). Endocytosis, phagosome, lysosome, regulation of autophagy, together with the categories of cell motility, cell cycle, and communication, are the largest subcategories in the Cellular Process category. Only a few transcripts were annotated to de novo biosynthesis of amino acids.

### Transcripts involved in carbohydrate metabolism

Annotations of most of the carbohydrate-active enzyme (CAZyme) transcripts were consistent using both the NR and the UniProt databases (Additional file 5: Table S5). Transcripts were annotated to encoding utilization of starch, hemicellulose, mannan, glycogen, other glucans, pectin, peptidoglycan, chitin, galactoside, raffinose, rhamnoside, and xanthan. Comparison of the transcript sequences to the CAZy database [25] using dbCAN, which employs a hidden Markov model [26], revealed more than 300 transcripts that were annotated to encoding one or more domains characteristic of CAZymes. The predicted CAZymes included one family of Auxiliary Activities, 11 families of Carbohydrate-Binding Module (CBM), 7 families of Carbohydrate Esterase (CE), 28 families of Glycoside Hydrolase (GH), 18 families of Glycosyl Transferase (GT), and 2 families of Polysaccharide Lyase (Table 1). Some transcripts were predicted to bind to peptidoglycan and chitin (annotated to CBM50), starch (CBM20, which has a granular starch-binding function), and xylan (CBM13). Multiple families of acetyl xylan esterase were represented in the transcriptome, together with other esterases. The majority of the CAZymes was associated with degradation of xylan (e.g., GH3 and GH43), starch (GH13, GH31), peptidoglycan (GH18, GH24, and GH25), and chitin (GH18) (Additional file 6: Table S6). Among the GT families, GT38, GT8, and GT4 were each represented by multiple transcripts. They are involved in the degradation of large branched glycan polymers and sugar metabolism. Some transcripts were annotated to encoding swollenin/expansin proteins (Additional file 6: Table S6), which do not have any enzyme activity but can enhance the CAZymes activities [27]. Transcripts encoding the enzymes involved in glycogen synthesis, such as UDP-Glc:glycogen glucosyltransferase, glycogen synthase kinase-3 beta, and 1,4-alpha-glucan-branching enzyme, were well presented (Table 1, Additional file 5: Table S5).

**Table 1:** The CAZymes families represented in the *Entodinium caudatum* transcriptome

Family	NO. transcripts	SP^a^	Note^a^
AA6	1	1	
CBM6	1		
CBM13	9		
CBM18	1		
CBM20	20		
CBM32	2		
CBM35	1		
CBM45	4		
CBM50^‡^	3	1	
CBM57	2	2	
CBM63	1	1	
CBM67	1		
CE1	21		
CE2	1		
CE3	1		
CE4	3		acetyl xylan esterase, deacetylase (chitin, chitooligosaccharide, peptidoglycan GlcNAc, peptidoglycan MurNAc)
CE7	10		
CE10^†^	23	7	
CE14	1		
GH2	6		
GH3	20	7	
GH5	2	2	
GH9	1		
GH13	33	14	
GH16	4	2	
GH18	8	6	chitinase, lysozyme
GH24	4	1	lysozyme
GH25	20	12	lysozyme
GH27	1		
GH28	1		
GH30	1	1	
GH31	12	3	
GH33	1	1	
GH38	2	1	
GH43	2	2	
GH53	1		
GH55	1		
GH74	1	1	
GH76	2	1	
GH77	1	1	
GH78	2		
GH84	6	6	N-acetyl β-glucosaminidase
GH87	4		
GH89	1		α-N-acetylglucosaminidase
GH93	6		
GH105	1	1	
GH125	3		
GT1	1		
GT2	38		
GT4	10		
GT5	2		UDP-Glc:glycogen glucosyltransferase, ADP-Glc:starch glucosyltransferase, NDP-Glc:starch glucosyltransferase, UDP-Glc:α-1,3-glucan synthase, UDP-Glc:α-1,4-glucan synthase
GT8	14		
GT10	1		
GT17	5		
GT20	3		
GT23	2		
GT28	1		
GT33	1		
GT35	1		glycogen or starch phosphorylase
GT41	1		
GT50	1		
GT69	2		
GT75	1		
GT83	1		
GT92	7		
PL8	1		
PL9	1		lyase (thiopeptidoglycan, pectate, exopolygalacturonate)

^a^ enzymes activities or substrates are indicated for the families that contain lysozyme enzymes and CAZymes that are involved in degradation of chitin and peptidoglycan or synthesis of glycogen

Furthermore, annotation against the NR and the Uniprot databases also identified genes involved in utilization of different sugars and their derivatives, including glucose, mannose, galactose, glucuronic acid, and ribose (Additional file 4: Tables S4 and Additional file 6: Table S6). Except for two genes (the genes encoding phosphoglucose isomerase and fructose-bisphosphate aldolase), all the genes of the Embden–Meyerhof–Parnas (EMP) pathway for glycolysis had corresponding transcripts. Transcripts involved in xylose degradation included those encoding D-xylose 1-dehydrogenase and (NADP+)- and NAD(P)H-dependent D-xylose reductases. One transcript was annotated to the pentose phosphate pathway, whereas some transcripts were annotated to pentose and glucuronate interconversions. Transcripts were well represented in the transcriptome that encode the degradative enzymes of N-acetylglucosamine (GlcNAc) and N-acetylmuramic acid (MurNAc), such as GlcNAc kinase, MurNAc-6-phosphate etherase (or lyase), and anhydro-GlcNAc kinase. Several transcripts were annotated to trehalose synthesis (e.g., trehalose 6-phosphate synthase) (Additional file 5: Table S5).

**Fig. 3:**
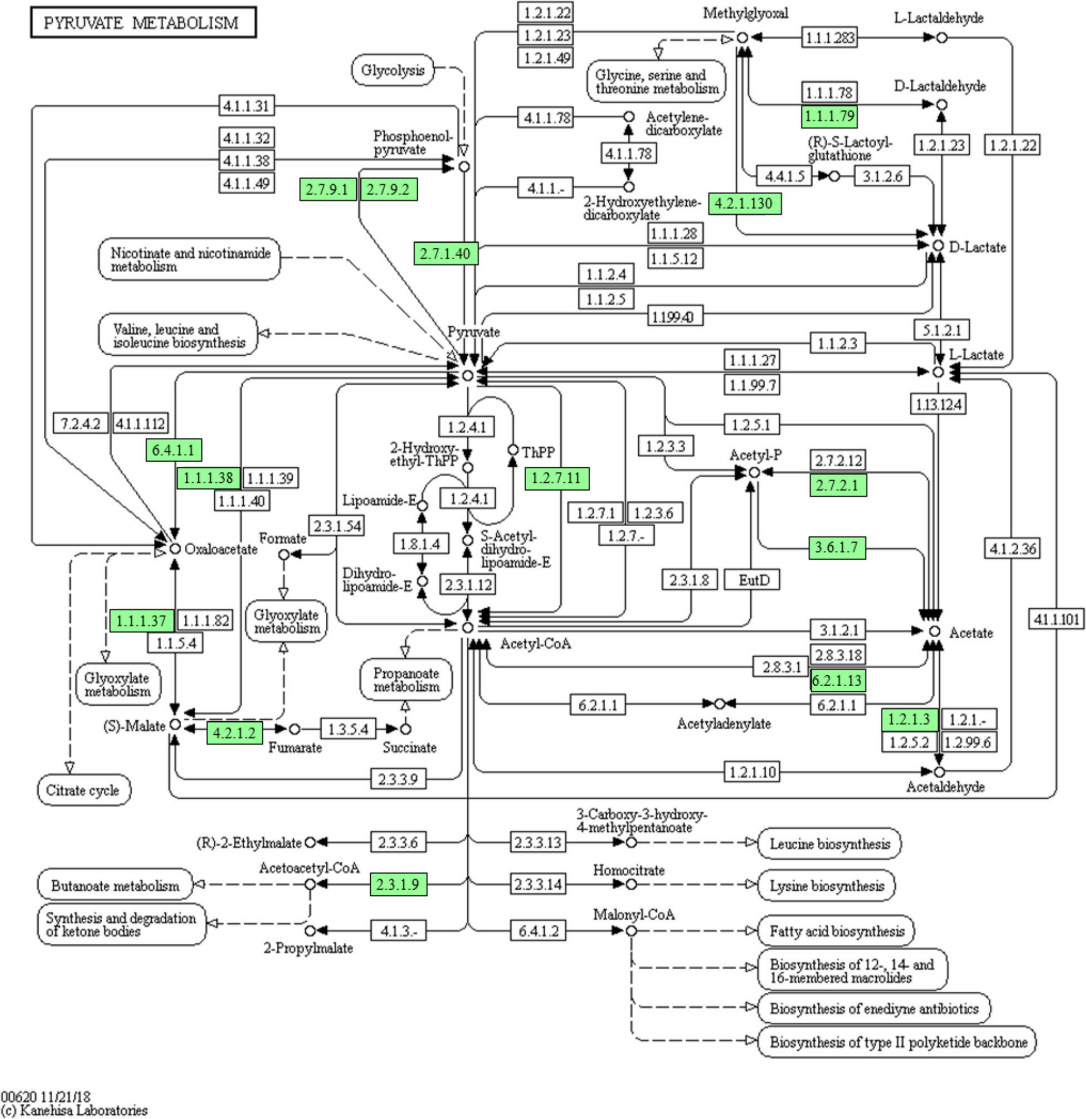
The pyruvate metabolism pathway. The pathway map was generated using KAAS https://www.genome.jp/tools/kaas/. The genes highlighted in green were identified in this study. The metabolic map was obtained from KEEG, which granted the permission to use this map in this article

Many transcripts were annotated to encoding enzymes involved in the fermentative processes from pyruvate to some of the fermentation products found in the rumen (Fig. 3). The acetate production pathway was represented by pyruvate dehydrogenase bypass (pyruvate metabolic process, GO:0006090) and acetate kinase, with the phosphotransacetylase not being represented. Except for butyryl–CoA dehydrogenase, all the enzymes of the butyrate production pathway were represented (pyruvate carboxylase, acetyl-CoA C-acetyltransferase, 3-hydroxybutyrate dehydrogenase, enoyl-CoA hydratase (crotonase), phosphate butyryltransferase, and butyrate kinase). No transcript was found for the acrylate pathway or propanediol pathway of propionate production. Except for fumarase, all the enzymes involved in succinate production were represented (phosphoenolpyruvate carboxylase, malate dehydrogenase, and fumarate reductase). Some transcripts were annotated to D-lactate dehydrogenase and lactate biosynthetic process (GO:0019249). Some transcripts were found to code for dehydrogenase of aldehyde and alcohol. Formaldehyde dehydrogenase was represented by three transcripts, but no transcript encoded pyruvate formate lyase. Two types of hydrogenases were found: ferredoxin hydrogenase and iron hydrogenase.

### Transcripts involved in protein degradation

Ruminal ciliates engulf large amounts of other microbial cells in the rumen, and *E. caudatum* is notorious for its high bacterivory [28]. The *E. caudatum* transcriptome was compared to the MEROPS database (www.ebi.ac.uk/merops/) to identify putative peptidases (proteases, proteinases, and proteolytic enzymes) and inhibitors. The comparison revealed 615 putative proteinases (Table 2), and some of them were annotated to having a signal peptide, a transmembrane domain, or both. The putative proteinases were assigned to more than 60 families, and the four major catalytic types of peptidases (cysteine, metallo, aspartic, and serine) each were represented by a large number of transcripts. Among the annotated aspartic peptidases, family A01A had the most transcripts followed by A22A. These two subfamilies contain endopeptidases that are most active at acidic pH and membrane-inserted endopeptidases, respectively. Family C19, which is a group of ubiquitin-specific peptidases, was the largest peptidase family among the annotated cysteine peptidases, followed by C01A, which contains both papain endo- and exo-peptidases, and C02A and C54, which contain calcium-dependent calpain peptidases and endopeptidases, respectively, with specificity for glycyl bonds. Among the annotated metallopeptidase families, M08, which contains zinc metalloendopeptidases and its homologs with acidic pH optima, followed by M01, which is primarily aminopeptidases. Only two subfamilies of serine peptidases were annotated: S01A and S01B (both are serine endopeptidase). More than 40 transcripts were annotated to coding for peptidase inhibitors (Additional file 7: Table S7). The family I50B (inhibitor of C14) had the most transcripts, followed by I04 (inhibitors of serine and cysteine endopeptidases).

**Table 2:** Putative proteinase families predicted in the *Entodinium caudatum* transcriptome

Catalytic Type	Family	No. of Transcripts	No. of transcripts with SPs^a^	No. of transcripts with TMs^b^	No. of transcripts with both SPs and TMs
Aspartic (A)	A01A	27	10	12	7
	A01B	7		2	
	A02B	1	1		
	A22A	12			
	A22B	4		2	1
	A28	2		1	
	Total	52		17	8
Cysteine (C)	C01A	41	15	6	4
	C01B	2			
	C02A	34	2		
	C04	6			
	C12	3			
	C13	7	1	3	1
	C19	222	1	8	
	C23	2			
	C26	9	2	3	
	C40	8	2	1	1
	C48	28			
	C50	1			
	C54	32			
	C56	3			
	C59	1			
	C65	3			
	C67	1			
	C69	6			
	C78	1			
	C83	1			
	C85A	8			
	C87	1			
	C95	4	1		2
	C97	2			
	Total	422	35	21	8
Metallo (M)	M01	14	2		
	M03A	3		1	
	M03B	4			
	M08	34	10	5	3
	M12B	1			
	M14A	2			
	M14B	4			
	M14D	2			
	M15C	1			
	M15D	2		1	
	M16A	9		1	
	M16B	1			
	M16C	2			
	M16X	2			
	M18	1			
	M20C	1			
	M20X	2			
	M24A	6		2	
	M24B	1			
	M24X	4		1	
	M28A	1			
	M28X	1			
	M38	2			
	M41	1			
	M43A	1			
	M48A	2		2	
	M54	4			
	M56	1		1	
	M67A	3		1	
	M76	1			
	M79	4		3	
	Total	116	12	17	3
Mixed (P)	P01	1	1		
	Total	1	1		
Serine (S)	S01A	4			
	S01B	30			
	Total	34			
Total		615			

^a^ *SP* signal peptide. ^b^*TM* transmembrane domains

### Transcripts involved in signal transductions, phagocytosis, intracellular trafficking, and vesicular transport

**Fig. 4:**
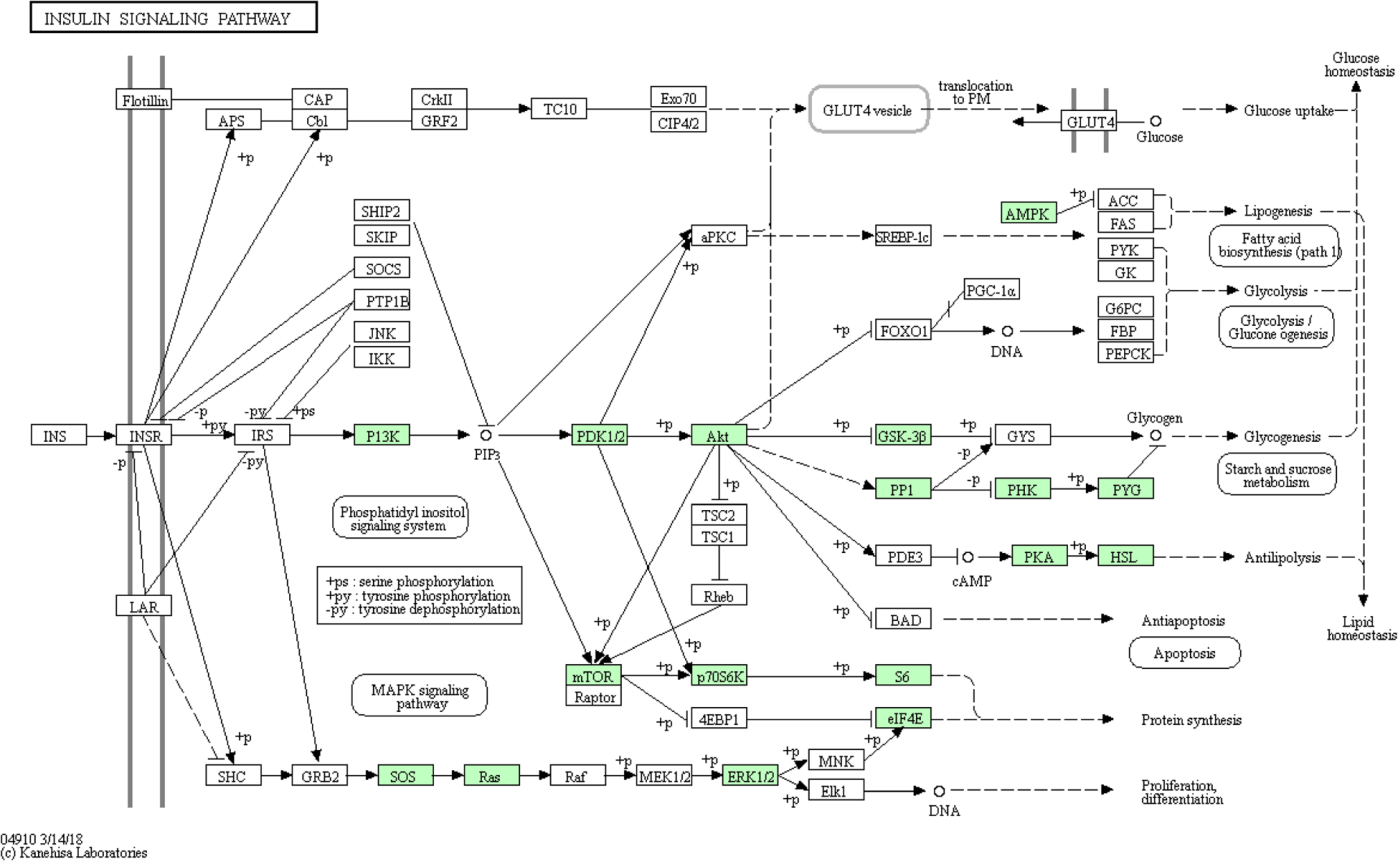
The insulin signal pathway. The pathway map was downloaded from www.genome.jp/kegg/pathway.html. The genes highlighted in green were identified in this study. The signaling pathway map is obtained from KEEG, which granted the permission to use this map in this article

Annotation using both the GO and KEGG databases revealed a large number of transcripts that were mapped to many different signaling pathways, including MAPK, mTOR, PI3K-Akt, AMPK, Wnt, calcium, and Hedgehog signaling pathways (Table 3 and Additional file 3: Table S3, Additional file 4: Table S4, and Additional file 8: Table S8). 14–3-3 proteins, which can bind to a multitude of functionally diverse signaling proteins, including kinases, phosphatases, and transmembrane receptors, were among the highly expressed (Additional file 2: Table S2). The transcriptome contained multiple transcripts annotated to genes of the insulin signaling pathway (Fig. 4).

**Table 3:** Signal transduction pathways predicted for in E. caudatum*

PATH ID	Pathway Name	No. of KOs
ko02020	Two-component system	1
ko04010	MAPK signaling	20
ko04012	ErbB signaling	10
ko04014	Ras signaling	19
ko04015	Rap1 signaling	11
ko04020	Calcium/calmodulin signaling	10
ko04022	cGMP-PKG signaling	13
ko04024	cAMP signaling	13
ko04064	NF-kappa B signaling	6
ko04066	HIF-1 signaling	9
ko04068	FoxO signaling	19
ko04070	Phosphatidylinositol signaling system	21
ko04071	Sphingolipid signaling	19
ko04072	Phospholipase D signaling	18
ko04075	Plant hormone signal transduction	5
ko04150	mTOR signaling	21
ko04151	PI3K-Akt signaling	25
ko04152	AMPK signaling	19
ko04310	Wnt signaling	9
ko04330	Notch signaling	3
ko04340	Hedgehog signaling	9
ko04350	TGF-beta signaling	6
ko04370	VEGF signaling	9
ko04371	Apelin signaling	19
ko04390	Hippo signaling	8
ko04630	JAK-STAT signaling	3
ko04668	TNF signaling	8
ko04062^a^	Chemokine signaling	13
ko04620^a^	Toll-like receptor signaling	7
ko04621^a^	NOD-like receptor signaling	14
ko04622^a^	RIG-I-like receptor signaling	5
ko04624^a^	Toll and Imd signaling	3
ko04625^a^	C-type lectin receptor signaling	12
ko04910^b^	Insulin signaling	18
ko04911^b^	Insulin secretion	4
ko04912^b^	GnRH signaling	12
ko04920^b^	Adipocytokine signaling	8
ko04922^b^	Glucagon signaling	10
ko04931^c^	Insulin resistance	14

*This table is a summary of the signal transduction pathways predicted based on both the GO and KEGG database. All the genes assigned to a GO signal transduction categories were further searched in the KEGG database to find their corresponding KO and pathways involved. All the pathways assigned to a KEGG signal transduction pathway category were listed, plus some other related pathways. ^a^, Immune system; ^b^, Endocrine system; ^c^, Endocrine and metabolic diseases

Many transcripts were annotated to phagocytosis, phagosome, lysosome, the process and structural and functional components that are involved in engulfment and digestion of microbial cells (Additional file 4: Table S4). The highly expressed GO terms included taxis (GO:0042330), cell motion (GO:0006928), cell mobility (GO:0048870), MKS complex (GO:0036038), membrane docking (GO:0022406), vesicle (GO:0031982), vesicle targeting (GO:0006903), vesicle-mediated transport (GO:0016192), tethering complex (GO:0099023), ESCRT complex (GO:0036452), clathrin-coated pit (GO:0005905), and retromer complex (GO:0030904) (Additional file 3: Table S3). Some transcripts were annotated to the phospholipase D signaling pathway (Additional file 8: Table S8), which is involved in regulating membrane trafficking, cytoskeletal reorganization, receptor-mediated endocytosis, exocytosis, and cell migration [29], and SNARE interactions in vesicular transport, which is involved in membrane fusion [30, 31], were also found (Additional file 4: Table S4).

### Transcripts involved in symbiosis and other activities

Some transcripts were annotated to coding for structures and activities involved in symbionts (Additional file 3: Table S3). These include interspecies interaction between organisms (GO:0044419), host cellular component (GO:0018995), host cell part (GO:0033643), other organism cell (GO:0044216), adhesion of symbiont to host (GO:0044406), symbiont-containing vacuole membrane (GO:0020005), development involved in symbiotic interaction (GO:0044111), growth involved in symbiotic interaction (GO:0044110), dissemination or transmission of organism from other organisms involved in symbiotic interaction (GO:0051821), multi-organism localization (GO:1902579), and movement in environment of other organism involved in symbiotic interaction (GO:0052192).

Some transcripts were annotated to enzymes or proteins involved in activities and features probably particularly unique to rumen ciliates (Additional file 3: Table S3). Many transcripts were annotated to encoding quenching of reactive oxygen species (e.g., GO:0004601, peroxidase activity; GO:0004784, superoxide dismutase activity), regulation of osmolarity (GO:0010118, stomatal movement), cell communication (GO:0007154), cilia and extracellular structure (GO:0030030, cell projection organization; GO:0043062, extracellular structure organization), localization (GO:0051234, establishment of localization; GO:0051235, maintenance of location; GO:0032879, regulation of localization), regulation of circadian rhythm (GO:0042752, GO:0042753), regulation of biological quality (GO:0065008), detection of stimulus (GO:0051606) and response to stimuli (GO:0006950, response to stress; GO:0006955, immune response; GO:0007610, behavior; GO:0009605, response to external stimulus; GO:0009607, response to biotic stimulus; GO:0009628, response to abiotic stimulus; GO:0009719, response to endogenous stimulus; GO:0042221, response to chemical stimulus; GO:0051716, cellular response to stimulus; GO:0051707, response to other organisms). Two transcripts were predicted to be involved in conjugation (GO:0000742 karyogamy involved in conjugation with cellular fusion). The dearth of transcripts annotated to conjugation is consistent with binary division being observed as the primary method of reproduction in *E. caudatum* [32].

### Comparison with the genomes of well-characterized ciliates

This is the first study to characterize the transcriptome of a rumen ciliate. To gain a glimpse of the metabolic features of this symbiotic ciliate, the transcriptome of *E. caudatum* was compared to the macronuclear genomes of *Paramecium tetraurelia* and *T. thermophila*, two free-living model aerobic ciliate species, with a focus on the CAZymes and peptidases. Only one-third of the transcripts of the *E. caudatum* displayed a moderate similarity with the genes of the two free-living model ciliates (Additional file 9: Table S9). Compared to the genomes of these two model ciliates, the *E. caudatum* transcriptome was enriched with numerous CAZymes (i.e., CBM13, CBM20, CE1, CE10, GH3, GH16, GH18, GT2 GT4, and GT8) and peptidases (C01A, C02A, C19, C26, C54, M01, and S01B).

## Discussion

The rumen is a luxurious environment for anaerobic ciliates because of the rich and consistent availability of substrates and preys (primarily bacteria) and stable temperature and pH (with some fluctuation but mostly less than half a pH unit). This transcriptomic study revealed many of the important features of *E. caudatum*, a common rumen ciliate species. Some of the features related to its metabolism and lifestyle in the rumen are discussed below with a focus on those implicated in the utilization of structural polysaccharides, nitrogen metabolism, and nitrogen utilization efficiency in ruminants. Some of the features help understand the niche and fitness of *E. caudatum* as a common rumen ciliate. We want to point that that the lack of transcripts annotated to certain enzymes or proteins does not necessarily reflect the lack of the corresponding genes because, to be conservative, we excluded from the bioinformatic analyses the transcripts that had ≤5× sequencing coverage or that were shared a greater than 90% sequence identity with non-protozoal sequences.

### Structural features

Ciliates are unicellular organisms, and they typically have extracellular structures to protect the cytoplasmic membrane. In *T. thermophila* and *P. tetraurelia*, the pellicle serves this purpose. Only one published study [33] has examined the surface structure of one species of rumen ciliate, *Isotricha intestinalis*. However, that study did not compare or relate the *Isotricha intestinalis* surface structure to that of the model ciliates. In two early studies, the surface of two rumen ciliates (*Epidinium ecaudatum* subsp. *caudatum* and *E. caudatum*) were referred to as pellicle but offered no description of the structure [34]. Considering the large numbers of transcripts annotated to extracellular structure components, such as cell periphery, extracellular organelles, extracellular matrices, and extracellular region parts, *E. caudatum* likely has an extracellular structure that is better examined using electron microscopy. Indeed, the extracellular surface structure of *E. caudatum* was clearly revealed by both scanning and transmission electron microscopy in a recent study [9]. A periplasmic space is probably also present between the cell surface structure and the cytoplasmic membrane, as indicated by the transcripts annotated to extracellular and periplasmic space in the *E. caudatum* transcriptome. In *T. thermophila*, the trimethylamine N-oxide reductase (TMAO) (TIGR02955) system was found as a periplasmic protein (http://ciliate.org/index.php/feature/details/TTHERM_00937640). Another periplasmic protein of *T. thermophila* is thiol:disulfide oxidoreductase, which is required for disulfide bond formation in proteins that are exported from the cytoplasm [35]. The periplasmic space of *E. caudatum* is likely a space for multiple activities that await further determination.

### Major substrates and metabolism

Carbohydrates, primarily polysaccharides, are the primary substrates for the rumen microbes, including *E. caudatum*. Mixed cultures of *E. caudatum* and other rumen microbes are maintained on feedstuffs consisting of starch, cellulose, and hemicellulose [9, 36]. In the *E. caudatum* transcriptome, the annotated CAZymes included amylases, hemicellulases, cellulases, and pectinases (including pectate lyase). Compared to the genomes of *T. thermophila* and *P. tetraurelia*, the transcriptome of *E. caudatum* has more genes encoding different CAZymes involved in xylan and starch hydrolysis. Given the much larger numbers of transcripts involved in starch utilization than those involved in the utilization of cellulose and hemicellulose, *E. caudatum* probably prefers starch, particularly granular starch as indicated by the high expression of CBM20 (binding to starch granules), over other carbohydrates as its major energy source. A recent study did show that *E. caudatum* engulfed starch granules and converted the digestion products to glycogen [37]. *E. caudatum* cells isolated and washed from the rumen showed limited abilities to hydrolyze xylan, carboxymethylcellulose (CMC), and cellulose Azure, but not microcrystalline cellulose [38]. The small numbers of GH transcripts annotated to cellulases and hemicellulases reflect the lack of diverse cellulases or hemicellulases. Future research can quantify the expression of the genes encoding these GHs and their functionality. Three of the transcripts encode swollenin/expansin-like proteins that are similar to swollenin/expansin found in the genome of *Entamoeba histolytica* [39], a protozoan parasite afflicting primates. Expansins are small proteins first discovered in plants, but they were also found in many microbes [27] and the eukaryotic metatranscriptome of the rumen of muskoxen [20, 40, 41]. They do not have hydrolytic activity, but they can bind to and loosen plant cell wall materials to aid fiber hydrolysis [27]. Given the presence of expansin-coding genes in the genome of *Entamoeba histolytica* [39], which has no known ability to degrade cellulose or hemicellulose, the finding of expansin transcripts in the transcriptome of *E. caudatum* is intriguing.

Glycogen is the main storage carbohydrate in *E. caudatum* [9, 37], and indeed transcripts encoding glycogen synthesis enzymes (e.g., UDP-Glc:glycogen glucosyltransferase) were identified. The hydrolysis and degradation of glycogen were evidenced by the transcripts encoding the glycogen phosphorylase and glycogen-debranching enzymes. Transcripts were found to be involved in synthesis (e.g., trehalose phosphate synthase) of alpha-trehalose, which can be used as an osmoprotectant by *Fabrea salina*, a hypersaline ciliate [42]. In *Saccharomyces cerevisiae*, trehalose is also a storage carbohydrate, a stabilizer and protector of membranes and proteins, a safety valve against damage caused by oxygen radicals, and a regulator of the glycolytic pathway [43]. Given the high osmolarity in the rumen fluid, trehalose likely serves as an osmoprotectant in *E. caudatum*. However, the possibility of trehalose to be a storage carbohydrate cannot be ruled out.

As a fermentative ciliate, *E. caudatum* ferments sugars to volatile fatty acids (VFA) and to produce ATP. As indicated by the transcripts involved in the EMP pathway and the pentose pathway, *E. caudatum* probably uses these two pathways to catabolize hexoses and pentoses, respectively. Acetate, butyrate, and propionate were the major VFA detected in the monocultures of *E. caudatum* [44, 45]. However, the monoculture contained prokaryotes of unknown species. No study has reported VFA production by axenic cultures of *E. caudatum.* The finding of transcripts encoding the enzymes involved in the fermentative formation of acetate and butyrate, although one enzyme of each of the pathways was not found, provided transcriptomic evidence for its fermentation profiles from pyruvate. The lack of any transcript annotated to the acrylate pathway or the propanediol pathway suggests that *E. caudatum* does not produce propionate. The high expression of aldehyde dehydrogenase and alcohol dehydrogenase genes also suggests the ability to produce ethanol as a fermentation product. *E. caudatum* was shown to utilize lactate [46], and this ability is corroborated by the lactate dehydrogenase transcripts. *E. caudatum* probably does not produce formate because no transcript encoded pyruvate formate lyase. No transcript was found to encode acetate:succinate CoA-transferase, the last enzyme mediating acetate formation in hydrogenosomes [47]. This corroborates the previous reports that *Entodinium* spp. lack hydrogenosomes [9, 48].

### Engulfment of other microbes and utilization of their macromolecules

*E. caudatum* is the most bacterivorous of the characterized ciliates in the rumen [28]. Many proteins are involved in phagocytosis that involves membrane trafficking and subsequent formation of phagolysosomes [49]. Not surprisingly, a large number of transcripts appeared to be involved in the physiological processes of phagocytosis, phagosome-lysosome trafficking, and regulation of autophagy. No transcript was annotated to mannose 6-phosphate receptor; thus, the lysosomal enzymes are probably transferred to lysosomes via the mannose-6-phosphate receptor-independent pathway(s) [50]. Also, the transcriptome of *E. caudatum* had a large number of transcripts encoding lysozyme, which were assigned to GH18, GH24, and GH25, with the latter two GH families exclusively containing lysozymes. These lysozyme transcripts corroborate the exceptionally high bacterivory of *E. caudatum* via digesting the peptidoglycan of the bacterial cell wall. Compared to the genomes of *T. thermophila* and *P. tetraurelia*, the transcriptome of *E. caudatum* has more genes encoding lysozyme, chitinase, and peptidases. This might be attributed to the long-term evolution in the presence of a high density of microbial cells. The transcripts encoding N-acetyl β-glucosaminidase and α-N-acetylglucosaminidase, both of which are lysosomal enzymes, and GlcNAc kinase, MurNAc-6-phosphate etherase, and anhydro-GlcNAc kinase suggest probable utilization of both the GlcNAc and MurNAc released from peptidoglycan hydrolysis mediated by the lysozyme. This premise is consistent with the degradation of the bacterial cell wall by *E. caudatum* monocultures, although the monoculture contained prokaryotes of unidentified species [51]. The discovery of chitinase transcripts indicated that *E. caudatum* engulfs and digests fungal cells, and the released fungal GlcNAc may be utilized like the bacterial GlcNAc that is released from the bacterial cell wall. Chitin degradation by protozoa, but not specifically *E. caudatum*, has indeed been reported [52–54]. Engulfment of fungal zoospores by *Entodinium* sp. has also been observed by scanning electron microscopy [32]. From a nitrogen utilization perspective, lysozyme can be inhibited to decrease the wasteful degradation of microbial proteins to improve nitrogen utilization efficiency in and decrease nitrogen excretion from ruminant livestock.

Many peptidase genes of the four main families were expressed at high levels, suggesting active degradation of the engulfed microbial proteins. Among the peptidases, C19 gene was expressed to the highest level. As a ubiquitin-specific peptidase, it is mainly involved in proteolysis in both the proteasome and the lysosome. It is not certain if the high expression of C19 gene reflects its role in the proteolysis of *E. caudatum* proteins or degradation of microbial proteins of the engulfed prey. The subfamily C01A gene also was highly expressed. This subfamily contains papain peptidases, including cathepsins that are lysosomal peptidases. In the transcriptome, cathepsin A, B, D, E, and F were represented. Cathepsin may play an important role in lysosomal degradation of microbial proteins. Three of the four highly expressed cysteine proteinase (C01A, C02A, C19, ad C48) were found to have a predicted signal peptide. These peptidases can be transmembrane proteins within the lysosome or extracellular peptidases. Cysteine proteases with signal peptides were found in *T. thermophila* [55], which secretes proteases [56, 57]. Given the ability to engulf microbial cells, it is intriguing that ciliates may also secrete peptidases. Future research is needed to determine if *E. caudatum* does secrete peptidases. It can be difficult, however, to distinguish the extracellular peptidases secreted from those discharged via the feed digestive vacuoles. Consistent with the rapid degradation and availability of free amino acids derived from microbial protein degradation, only a few transcripts were annotated to de novo synthesis of amino acids, which explains their dependence on bacterial protein as their main protein source [32]. However, small entodinia are often considered the most bacterivorous [3], and the dependence on preformed amino acids may explain the difficulty to grow *E. caudatum* in axenic cultures [9]. The major families of the peptidases may be targeted to inhibit or control rumen ciliates to improve nitrogen utilization efficiency in ruminants.

Both phagocytosis and feed vacuole movement entail membrane trafficking and recycling. The many transcripts annotated to these processes and lipid metabolism are consistent with that requirement. Numerous transcripts were annotated to being involved in nucleotide metabolism. These transcripts may reflect the complex processes required for the formation of macronucleus or the ability of *E. caudatum* to degrade and then utilize some of the DNA and RNA of engulfed prey. Because no other rumen microbes conduct phagocytosis, the key enzymes involved in the phagocytosis and the membrane trafficking processes are other potential targets to control rumen ciliates.

### Responses to external stimuli, symbiosis, and other features

Rumen ciliates are known to rapidly respond to external stimuli, including the availability of nutrients [58, 59]. Although transcripts of the common signal transduction pathways are expected as the essential markers of chemotaxis and other responses to external stimuli, it is surprising that the *E. caudatum* transcriptome was represented by nearly 40 different signaling pathways. Signal transduction mediated by tyrosine kinases and serine/threonine kinase is important to phagocytosis in higher eukaryotes [60], and both kinases were represented by many transcripts in the *E. caudatum* transcriptome. Few studies have investigated the signal transductions in rumen ciliates. Diaz et al. [61] reported the first study that demonstrated the presence and function of PIK3-Akt and the calcium/calmodulin signaling pathways in *E. caudatum*. Future research may identify signaling pathways that are unique to *E. caudatum* and other rumen ciliates as potential targets for ciliate control in ruminants.

Rumen ciliates produce hydrogen, thereby forming a positive association with methanogens [62, 63]. Unlike other ruminal ciliates (e.g., species of *Epidinium*, *Isotricha*, and *Dasytricha*), *E. caudatum* contains no hydrogenosomes but does contain mitosomes [48, 64]. Malic enzyme, which is found in mitochondria, hydrogenosomes, and mitosomes, was represented in the *E. caudatum* transcriptome. Multiple transcripts were annotated to mitochondria. Because all the three types of organelles are related phylogenetically [65], the transcripts representing malic enzyme and mitochondria probably reflect the presence of mitosomes, rather than hydrogenosomes, in *E. caudatum.* Nevertheless, the revelation of iron hydrogenases in the *E. caudatum* transcriptome underpins hydrogen production by *E. caudatum.*

Rumen ciliates are assumed to be able to use free oxygen, thereby facilitating the anaerobiosis required for high fiber degradability and fermentation by the strictly anaerobic fibrolytic bacteria and for methanogenesis by archaea. The higher redox potential observed in defaunated than in faunated rumen also suggests the oxygen-scavenging ability of ruminal ciliates [66]. Following washing to remove bacteria, mixed rumen ciliates were shown to consume oxygen [10], and that ability was hypothetically attributed to the ciliates harboring hydrogenosomes, such as holotrichs and some entodiniomorphs [10, 32]. No study has tested if *E. caudatum* can consume oxygen. In the transcriptome, however, multiple transcripts were annotated to NADH dehydrogenase and the electron-transport chain, including cytochrome b5 and its reductase, and the transcripts showed similarity to the genes of *Stylonychia lemnae*, a free-living aerobic ciliate. Cytochrome c also had corresponding transcripts. In addition, multiple transcripts were annotated to peroxidases (NADH, glutathione, and thioredoxin peroxidases), suggesting a potential ability, probably very limited, to detoxify hydrogen peroxide. Transcripts annotated to superoxide dismutase related to that of *Salpingoeca rosetta*, a flagellated eukaryote, was also found. Nitrate reductase was represented by some transcripts and some of the above proteins, including NADH dehydrogenase and cytochromes, are also involved in nitrate reduction. Future research is needed to experimentally verify if *E. caudatum* can actually utilize free oxygen and/or nitrate as an electron acceptor to conserve energy.

Rumen ciliates were shown to have endosymbionts [67, 68], and *E. caudatum* requires some unknown prokaryotic symbionts for its survival [9]. *E. caudatum* also appeared to harbor specific bacteria, mostly members of phylum *Proteobacteria*, and these putative symbionts were similar between single *E. caudatum* cells isolated from monocultures maintained for several years in the laboratory and those isolated from fresh rumen fluid [69]. The finding of multiple transcripts annotated to symbiosis supports the symbiotic relationship between *E. caudatum* and some prokaryotes. As mentioned above, the transcriptome only had a few transcripts involved in de novo biosynthesis of amino acids or nucleosides. Although *E. caudatum* can obtain amino acids from proteolysis of microbial protein, the symbionts may also provide amino acids and other essential nutrients such as vitamins and other growth factors. Further research is needed to identify the *E. caudatum* symbionts and their metabolic relationships.

In summary, the transcriptome of *E. caudatum* revealed some of its features with respect to the substrate spectrum, metabolism and fermentation products, potential symbiosis, and oxygen consumption and tolerance. A number of genes that are important to *E. caudatum* but not to other members of the rumen microbiota, such as lysozyme, peptidases, and calcium-dependent protein kinases, the latter of which is expressed only in certain protozoa but not animals [70], may be targeted to develop specific inhibitors to control rumen ciliates to improve nitrogen utilization efficiency. Not all the transcripts can be described and discussed, and some transcripts were annotated to matching non-ciliate genes. Although precautionary steps were taken to remove contamination from other microbes by washing the *E. caudatum* cells before RNA isolation and removing RNA of other microbes bioinformatically, the transcriptome might still contain RNA sequences from other microbes. Equally plausible, these transcripts could also result from horizontal gene transfers from prokaryotes to rumen ciliates, which have been repeatedly documented [20, 40, 41]. Future genome sequencing will allow confirmation of transcripts of uncertain origin. The transcriptome data will also be valuable to help the assembly and annotation of genome sequences of rumen ciliates. They can also be further analyzed to address specific questions such as the ability to synthesize and the requirement for specific growth factors and circadian rhythm regulation of feeding and activities, both of which have been reported in some rumen protozoa [71–73].

## Conclusions

This is the first transcriptomic study of a single species of rumen ciliates. The transcriptome reveals the substrate spectrum, fermentation pathways, ability to respond to various biotic and abiotic stimuli, and other physiological and ecological features of *E. caudatum*. The high-level expressions of the genes involved in the lysis and degradation of microbial cells highlight the dependence of *E. caudatum* on engulfed rumen microbes for its survival and growth. These genes may be targeted to specifically control the activities and growth of *Entodinium* species in the rumen to help improve nitrogen utilization by ruminants. The transcriptome can also aid in future genomic studies of *E. caudatum* and other related rumen ciliates.

## Methods

### Strain, RNA extraction, and sequencing

Cells of *E. caudatum* MZG-1 were collected from a clonal monoculture of *E. caudatum* that was initially established from a single cell isolated from the rumen of gerenuk [36]. It was kindly given to us by Dr. Dehority (deceased). This monoculture does not have detectable fungus. Frozen stock cultures of *E. caudatum* MZG-1 were cryopreserved at − 80 °C and have been used in a number of studies [9, 69, 74, 75]. The *E. caudatum* MZG-1 monoculture was fed a mixed feed containing ground wheat grain, ground alfalfa, and ground grass hays and maintained in SP medium [9]. The feeding and transfer procedures were conducted under a continuous stream of CO_2_ to protect the ciliate cells from exposure to oxygen. Total RNA was isolated from an actively growing *E. caudatum* MZG-1 monoculture after six hours of incubation at 39 °C after transferring to fresh SP medium containing the mixed feed. Total RNA was extracted using the Ribozol RNA extraction reagent (Amresco, Inc., Solon, OH) and then cleaned up using the RNeasy® mini kit according to the manufacturer’s instructions (Qiagen, Inc., Valencia, CA). mRNA was enriched using the Oligo Direct mRNA Mini Kit (Qiagen). One library was constructed for 2 × 100 paired-end sequencing from the mRNA and then sequenced following the manufacturer’s protocol on an Illumina HiSeq 2000 system.

### Sequencing data processing, assembly, and gene annotation

The sequencing data were assembled using Trinity [23]. All the resulting contigs with a length less than 200 bp were discarded prior to further analyses. The coverage of the assembled contigs was estimated using genomecov (http://bedtools.readthedocs.io/en/latest/content/tools/genomecov.html) in -bga format. The assembled contigs were compared to the non-redundant (NR) protein database of the GenBank (https://www.ncbi.nlm.nih.gov/) and the Uniprot database (http://www.uniprot.org/) using BLASTX with a cutoff e-value less than 1e-5. Because the monoculture was not axenic and contained bacteria and archaea, the resulting annotation results were screened for prokaryotic genes. Singleton contigs and any contigs that had a sequencing coverage of less than 5× and that shared a greater than 90% sequence identity with non-protozoal sequences in the public databases were filtered out. Uncertain sequences (with an e-value ≥1E-10, no similarity to any eukaryotic genes in the first five hits in sequence comparison) were also discarded because they might be transcripts from other rumen microbes that remained after the decontamination (even though they might be genes transferred from other rumen microbes).

Protein domains were predicted using Pfam (http://pfam.xfam.org/). Putative proteases were predicted using the online server of the MEROPS protease database (http://merops.sanger.ac.uk/index.htm). Putative CAZymes were predicted using dbCAN, which employs a hidden Markov model [26], against the CAZy database [25]. The contigs were translated to amino acid sequences using TranslatorX [76] using the ciliate nuclear genetic codes [77] and then subjected to prediction of signal peptides and transmembrane domains using the SignalP 4.0 web server (http://www.cbs.dtu.dk/services/SignalP/) and the TMHMM 2.0 web server (http://www.cbs.dtu.dk/services/TMHMM/), respectively. The transcriptome of *E. caudatum* MZG-1 was also compared to the genome sequences of two model ciliates, *P. tetraurelia* and *T. thermophila*, using BLASTX with a cutoff of 1E-10 to identify shared genes. Gene Ontology (GO) annotations of the transcripts were done using the WEGO web server (http://wego.genomics.org.cn/cgi-bin/wego/index.pl) [78].

The NR annotations of the transcripts were also imported to MEGAN5 [79, 80] to predict their COG functional categories and mapped to metabolic pathways using the COG database [81]. Metabolic pathways were reconstructed using KAAS (KEGG Automatic Annotation Server for ortholog assignment and pathway mapping, https://www.genome.jp/tools/kaas/).

## Supplementary information

**Additional file 1: Table S1.** Summary of the transcriptome of *E. caudatum. (DOCX 14 kb)*

**Additional file 2: Table S2.** Most highly expressed mRNAs in *E. caudatum* transcriptome.

**Additional file 3: Table S3.** GO functional classification at subsystem_levels 1 to 3.

**Additional file 4: Table S4.** Predicted metabolic pathways of *E. caudatum* based on KEGG.

**Additional file 5: Table S5.** The transcripts related to carbohydrate metabolism.

**Additional file 6: Table S6.** The CAZymes represented in the *E. caudatum* transcriptome.

**Additional file 7: Table S7.** Putative proteinase inhibitors identified in the *E. caudatum* transcriptome.

**Additional file 8: Table S8.** Putative signal transduction pathways predicted from the *E. caudatum* transcriptome.

**Additional file 9: Table S9.** Comparison of CAZymes and peptidases of *E. caudatum* transcriptome to the genomes of two model ciliates.

## Data Availability

The raw Illumina sequences have been deposited in the Sequence Read Archive of GenBank under the admission number GHEK00000000. The version described in this paper is the first version, GHEK01000000.

## Abbreviations

AMPK: 5′ adenosine monophosphate-activated protein kinase
CAZy: Carbohydrate-active enzymes database
CAZymes: Carbohydrate-active enzymes
CBM: Carbohydrate-binding module
CE: Carbohydrate esterase
CoA: Coenzyme A
COG: Clusters of orthologous group
EMP: Embden–Meyerhof–Parnas
ESCRT: Endosomal sorting complexes required for transport
EST: Expressed sequence tag
GH: Glycoside hydrolase
GlcNAc: N-acetylglucosamine
GO: Gene ontology
GT: Glycosyltransferase
HGH: Horizontal gene transfer
KEGG: Kyoto Encyclopedia of Genes and Genomes
KO: KEGG orthology
MAPK: Mitogen-activated protein kinase
MEROPS: The peptidase database
mTOR: Mammalian target of rapamycin
MurNAc: N-acetylmuramic acid
NDPK: Nucleoside-diphosphate kinase
NGS: Next-generation sequencing
NR: Non-redundant
Pfam: Protein families
PI3K-Akt: Phosphoinositide-3-kinase and protein kinase B
PL: Polysaccharide lyase
PPDK: Pyruvate phosphate dikinase
SAGE: Serial analysis of gene expression
TCA: Tricarboxylic acid cycle
TMAO: Trimethylamine N-oxide reductase
TOR: Target of rapamycin
TORC: Target of rapamycin complex
UDP-Glc: Uracil-diphosphate glucose
VFA: Volatile fatty acid
WEGO: Web gene ontology annotation plot
