# Supplementary material for: The transcriptome of the rumen ciliate Entodinium caudatum reveals some of its metabolic features
Source: BMC Genomics. 2019 Dec 21;20:1008. doi: 10.1186/s12864-019-6382-x (PMC6925433; doi:10.1186/s12864-019-6382-x)
Supplement: Supplementary file 1 — Additional file 1: Table S1. Summary of the transcriptome of E. caudatum. (DOCX 14 kb) [file 12864_2019_6382_MOESM1_ESM.docx]

Suppl Table S1. Summary of the transcriptome of *E. caudatum.*

|  | Counts |
| --- | --- |
| Raw reads | 58,125,830 |
| After joining of paired reads | 29,062,915 |
| After quality filtering and removal of putative prokaryotic sequences | 21,549,192* |
| Total transcripts | 33,546 |
| GC % | 26.8 |
| Average length | 759 |
| N50 | 596 |
| Greatest coverage | 347,550 |
| Average coverage | 183 |
| Average contigs per transcript | 1,473 |
| Distribution of length of transcripts (LT) |  |
| 200 bp < LT <= 300 bp | 4,086 |
| 300 bp < LT <= 400 bp | 4,959 |
| 400 bp < LT <= 500 bp | 4,278 |
| 500 bp < LT <= 600 bp | 3,619 |
| 600 bp < LT <= 700 bp | 3,017 |
| 700 bp < LT <= 800 bp | 2,473 |
| 800 bp < LT <= 900 bp | 1,987 |
| 900 bp < LT <= 1,000 bp | 1,649 |
| 1,000 bp < LT <= 1,500 bp | 4,601 |
| 1,500 bp < LT <= 2,000 bp | 1,682 |
| 2,000 bp < LT <= 3,000 bp | 887 |
| 3,000 bp < LT <= 4,000 bp | 216 |
| 4,000 bp < LT <= 5,000 bp | 48 |
| 5,000 bp < LT <= 6,000 bp | 30 |
| 6,000 bp < LT <= 7,000 bp | 6 |
| 7,000 bp < LT <= 8,000 bp | 6 |
| 8,000 bp < LT <= 11,000 bp | 2 |
| Contigs without hit in the public database used in this study | 18,238 |
| Contigs with NR hits | 15,724 |
| Contigs with UniProt hits | 15,261 |
| Contigs with GO hits | 12,652 |
| Contigs with signal peptide | 1,044 |
| Contigs with transmembrane domain | 11,393 |

* About half of the removed transcripts were <300 bases, and most of them had no homologous sequences in any of the public databases searched in this study.
